# Symptom Burden and Complexity in the Last 12 Months of Life among Cancer Patients Choosing Medical Assistance in Dying (MAID) in Alberta, Canada

**DOI:** 10.3390/curroncol29030135

**Published:** 2022-03-03

**Authors:** Linda Watson, Claire Link, Siwei Qi, Andrea DeIure, K. Brooke Russell, Fiona Schulte, Caitlin Forbes, James Silvius, Brian Kelly, Barry D. Bultz

**Affiliations:** 1 Cancer Care Alberta—Alberta Health Services, Calgary, AB T2S 3C3, Canada; claire.link@albertahealthservices.ca (C.L.); siwei.qi@albertahealthservices.ca (S.Q.); andrea.deiure@albertahealthservices.ca (A.D.); bdbultz@ucalgary.ca (B.D.B.); 2 Faculty of Nursing, University of Calgary, Calgary, AB T2N 1N4, Canada; 3 Department of Psychology, University of Calgary, Calgary, AB T2N 1N4, Canada; kbrussel@ucalgary.ca; 4 Department of Oncology, Division of Psychosocial Oncology, University of Calgary, Calgary, AB T2N 1N4, Canada; fsmschul@ucalgary.ca (F.S.); caitlin.forbes@ucalgary.ca (C.F.); brian.kelly@newcastle.edu.au (B.K.); 5 Cumming School of Medicine, University of Calgary, Calgary, AB T2N 1N4, Canada; james.silvius@albertahealthservices.ca; 6 Provincial Seniors Health and Continuing Care, Alberta Health Services, Calgary, AB T2W 1S7, Canada; 7 School of Medicine and Public Health, University of Newcastle, Newcastle, NSW 2308, Australia

**Keywords:** Medical Assistance in Dying, medically assisted death, Medical Aid in Dying, MAID, symptom burden, symptom complexity, symptom management, Patient-Reported Outcomes

## Abstract

Background: In 2019, cancer patients comprised over 65% of all individuals who requested and received Medical Assistance in Dying (MAID) in Canada. This descriptive study sought to understand the self-reported symptom burden and complexity of cancer patients in the 12 months prior to receiving MAID in Alberta. Methods: Between July 2017 and January 2019, 337 cancer patients received MAID in Alberta. Patient characteristics were descriptively analyzed. As such, 193 patients (57.3%) completed at least one routine symptom-reporting questionnaire in their last year of life. Mixed effects models and generalized estimating equations were utilized to examine the trajectories of individual symptoms and overall symptom complexity within the cohort over this time. Results: The results revealed that all nine self-reported symptoms, and the overall symptom complexity of the cohort, increased as patients’ MAID provision date approached, particularly in the last 3 months of life. While less than 20% of patients experienced high symptom complexity 12 months prior to MAID, this increased to 60% in the month of MAID provision. Conclusions: Cancer patients in this cohort experienced increased symptom burden and complexity leading up to their death. These findings could serve as a flag to clinicians to closely monitor advanced cancer patients’ symptoms, and provide appropriate support and interventions as needed.

## 1. Introduction

Patients with cancer frequently experience acute and chronic symptoms and concerns across physical and psychosocial domains, caused by the impacts of their disease or treatment(s) [1]. An advanced cancer diagnosis is often accompanied by decisions regarding additional cancer therapies and the reality of what end-of-life care may look like once treatment options for extension of life have been exhausted [2]. The literature suggests that advanced cancer is often associated with suffering [3,4,5]. Suffering can be multidimensional and related to physical symptoms, such as pain and fatigue, but also encompasses psychological distress, existential concerns, and social–relational concerns [3,4,5]. Such suffering experienced by patients living with advanced cancer can manifest into the desire for death. This can range from wishing for death to occur sooner than it would naturally, to actively seeking medically assisted death [6,7]. Recognizing the suffering that patients with serious illness often experience, and understanding that some patients may have the desire for death, many jurisdictions and countries have developed legislation around medically assisted death. In Canada, this is referred to as Medical Assistance in Dying (MAID).

### 1.1. Medical Assistance in Dying in Canada

In 2016, the Canadian government passed Bill C-14, which decriminalized MAID [8]; the legislation was updated in March 2021 (Bill C-7) to remove one eligibility criterion [9]. The current criteria requires that the individual be an adult, capable of making their own health care decisions, with a “grievous or irremediable medical condition” [9]. Additionally, the condition must be “a serious illness, disease or disability” [9], the individual must be in an irreversible “advanced state of decline” [9] and be experiencing “unbearable physical or mental suffering from [their] illness that cannot be relieved under conditions that [they] consider acceptable” [9]. An advanced cancer diagnosis often meets these requirements, and in 2019, 67.2% of people who received MAID in Canada cited cancer as their main condition [10]. In Canada, written requests for MAID must be submitted to a practitioner and determined to have been made voluntarily [9]. While in other jurisdictions, such as the United States, medically assisted death often involves prescription medication that is self-administered by a patient who meets the criteria [11,12], MAID in Canada is almost always administered by a trained practitioner who is present at the time of the patient’s death [9,13].

As MAID legislation is still relatively new in Canada, much is unknown about patients who proceed with it. A recent study of individuals who were provided MAID in Ontario identified physical and/or psychosocial suffering as the primary reason, as opposed to socioeconomic factors [12]. Examining the specific self-reported symptoms of MAID patients could have important practice implications, while also informing the broader literature on the topic. In Alberta, 70% of patients who were provided MAID between 2017 and 2018 had a cancer diagnosis [14], suggesting that an in-depth look, specifically at cancer patients and their symptoms, may provide important provincial findings.

### 1.2. Patient-Reported Outcomes in Cancer Care Alberta

Cancer Care Alberta (CCA) is the provincial ambulatory oncology program in Alberta, providing publicly funded cancer care services through a provincial network of 17 ambulatory facilities. Alberta is home to 4.4 million people [15], with an expected annual cancer incidence of 23,424 by 2023 [16]. CCA supports the routine collection of Patient-Reported Outcomes (PROs) information through in-person clinical workflows [17]. Patients complete the provincial PROs questionnaire on paper at clinic appointments. It includes two standardized measures: the Edmonton Symptom Assessment System—Revised (ESAS-r), and the Canadian Problem Checklist (CPC) [18]. Together, the symptoms and concerns reported by the patient are used to generate a symptom complexity score from a unique validated algorithm [19]. The PROs questionnaire is used with all patients, including those with advanced cancer, and as such, the symptoms and concerns of specific patient cohorts can be tracked over time. In this descriptive study, we used PROs data to examine the symptoms and overall symptom complexity trajectory of a cohort of cancer patients who received MAID in Alberta, Canada. This is the first of several studies currently underway within CCA, with the goal of better understanding the experience of cancer patients who request and receive MAID.

## 2. Materials and Methods

### 2.1. Study Design

This descriptive retrospective cohort study utilized administrative data from CCA’s electronic medical records, chart audits, and the Alberta Cancer Registry. The study involved confidential patient data and was approved by the Health Research Ethics Board of Alberta’s Cancer Committee.

### 2.2. Sample and Data Collection

The study sample consisted of cancer patients who were provided MAID in Alberta between July 2017 and January 2019. We collected baseline characteristics including age at MAID provision, sex, tumour group, median neighborhood-level income (based on postal code linkage) [20] and Charlson Comorbidity Index (CCI), based on conditions present in the 12 months prior to the MAID provision date [21,22,23]. The provision date refers to when the MAID practitioner carried out the procedure and is identical to the patient’s date of death. Accordingly, age at MAID provision is the patient’s age at death. We used a modified version of the CCI to exclude cancer and associated metastases as contributing factors, as all patients had a cancer diagnosis. Baseline characteristics were chosen based on the availability of data routinely collected in Alberta. We also collected the date that patients signed their written MAID requests (“request date”).

A subgroup of cancer patients who received MAID within the time period also completed at least one PROs questionnaire in the 12 months prior to their death. PROs data were collected via chart audits in order to examine the self-reported symptoms, concerns and associated symptom complexity level of these patients.

### 2.3. Measures

#### 2.3.1. Patient-Reported Outcomes Questionnaire Components

**Figure A1:**
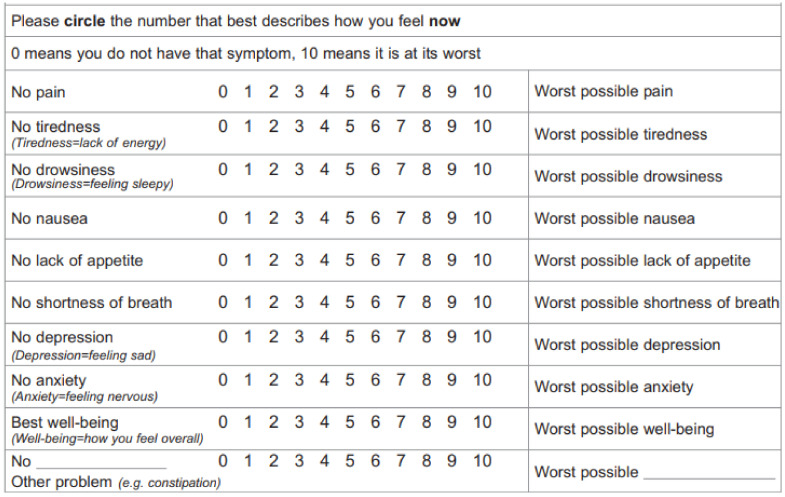
Edmonton Symptom Assessment System—Revised (ESAS-r). Used routinely throughout CCA as part of the routine Patient Reported Outcomes (PROs) questionnaire. Reproduced with permission from Sharon Watanabe, Edmonton Symptom Assessment System Revised (ESAS-r) [44].

**Figure A2:**
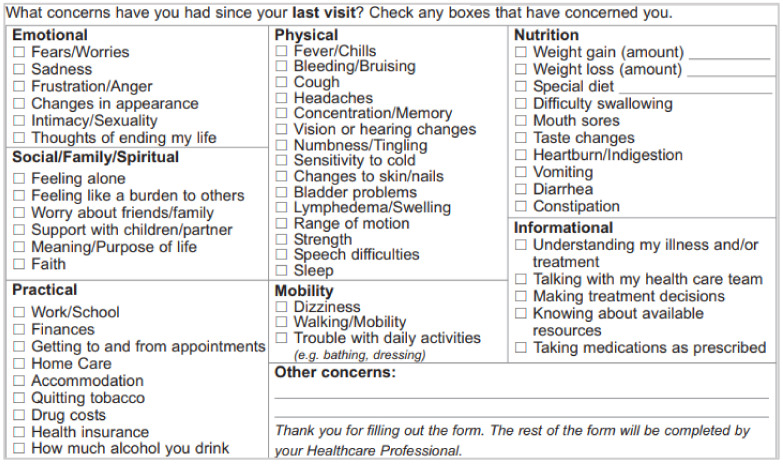
Modified 54-item version of the Canadian Problem Checklist (CPC). Version used routinely throughout CCA as part of the routine Patient Reported Outcomes (PROs) questionnaire. Modified with permission from Barry D. Bultz and Margaret I. Fitch, Canadian Problem Checklist © 2012 [25].

**Figure A3:**
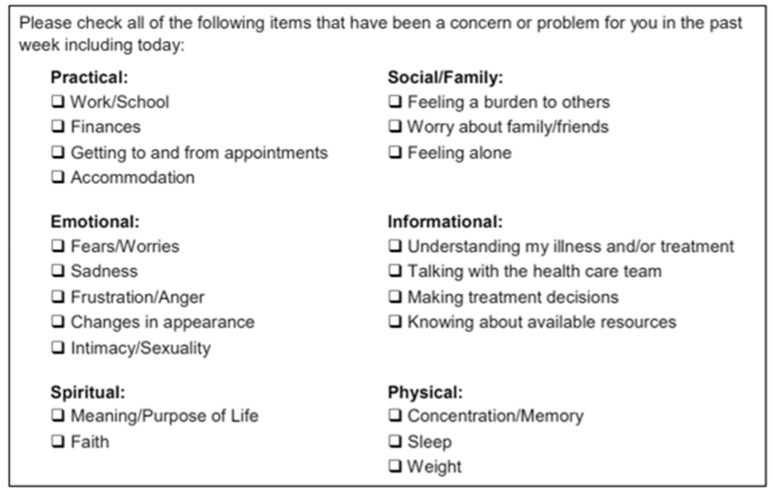
Original 21-item version of the Canadian Problem Checklist (CPC). The modified 54-item version used throughout CCA is based on this original version. Reproduced with permission from Barry D. Bultz and Margaret I. Fitch, Canadian Problem Checklist © 2012 [25].

The ESAS-r is a validated self-report questionnaire that measures nine symptoms: pain, tiredness, drowsiness, nausea, lack of appetite, shortness of breath, depression, anxiety, and well-being. Symptoms are rated from 0 to 10, with 0 indicating “None” or “Good” and 10 indicating “Worst”. The ESAS-r was originally developed for advanced cancer patients and offers ample evidence of validity for use with this population [24]. The CPC is a self-report checklist designed to identify common concerns that cancer patients experience across different domains [25]. Throughout CCA, a modified version of the original 21-item checklist is used. This modified version includes 54 items across seven domains: emotional, social/family/spiritual, practical, physical, mobility, nutrition, and informational [17]. The ESAS-r, modified CPC, and original CPC can be found in Appendix A (Figure A1, Figure A2 and Figure A3, respectively).

#### 2.3.2. Symptom Complexity Level

**Figure 1:**
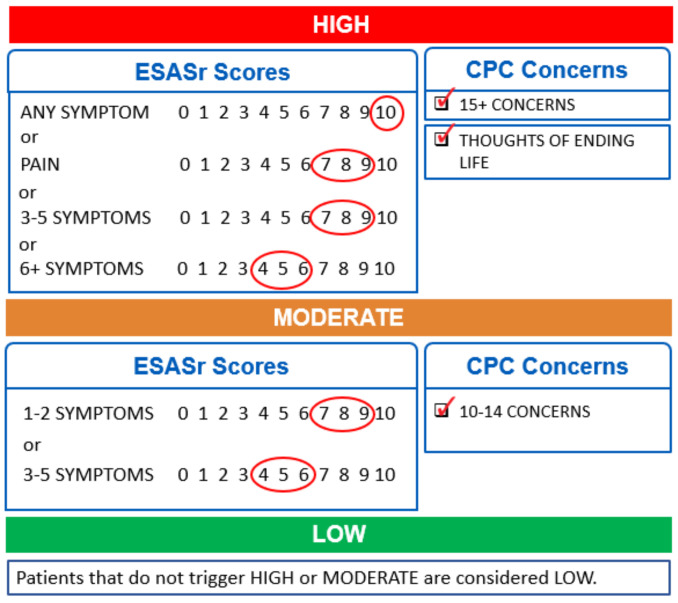
Criteria for each level of a 3-level validated PROs symptom complexity algorithm. Only one criterion in a level needs to be met to trigger that complexity score.

The ESAS-r and CPC results were used to determine a symptom complexity score for each PROs questionnaire, based on an original validated algorithm created within CCA [19]. The algorithm considers the unique combination of symptoms and concerns a patient has identified on a single questionnaire and assigns a symptom complexity score (low/green, moderate/yellow or high/red) for each encounter. The criteria triggering each complexity level are shown in Figure 1. Only one criterion in a level needs to be met to trigger that level’s complexity score.

### 2.4. Statistical Analyses

Descriptive statistics (mean, standard deviation, interquartile range, and frequency) were used to examine patient characteristics, symptom prevalence and symptom complexity. Chi-squared tests (for categorical variables) and independent t tests (for continuous variables) were used to test for differences between the subgroups with and without PROs. ESAS-r symptom scores were arranged based on the month the questionnaire was completed prior to the MAID provision date. Each month was coded in decreasing order from 12 to 1, with 12 indicating questionnaires completed 12 months prior to MAID provision and 1 indicating questionnaires completed within the same month as MAID provision. All questionnaires were included unless a patient had multiple within the same month, in which case the questionnaire with higher symptom scores was selected, in keeping with the approach followed in studies with similar methods [26]. To estimate longitudinal changes of ESAS-r scores in the 12 months prior to MAID, the primary analysis used a mixed effects model (MEM) for repeated measures [27]. The MEM allows for the timing and number of assessments to differ across patients and for the inclusion of time-varying covariates, and appropriately adjusts variance estimates for the correlation of repeated observations from the same patient [28]. With this type of real-world data involving morbidity and/or mortality, there will always be concerns about missing data [27,29]. However, in keeping with previous studies using a similar method, we assumed that data were missing at random, and therefore all available data were included [29].

Baseline variables potentially related to symptom scores were selected a priori based on data availability and literature, and are described above. Model selection was based on Akaike’s Information Criterion (AIC). The trend of symptom complexity level was modelled using Generalized Estimating Equations (GEE), as the outcomes were structured as ordinal (low, moderate and high complexity). We used the GEE approach to consider within-subjects variability and account for the correlated data resulting from repeated measurements across different time points and multiple observations of the same individual [27]. As the main effects of baseline characteristics were not of interest, only the main effect of time is reported (measured in months). We also calculated the median and interquartile range of the time, in months, from MAID request dates to provision dates. Data were exported into SPSS Version 25.0 (Chicago, IL, USA) for analysis and statistical significance was set a priori at *p* < 0.05.

## 3. Results

### 3.1. Study Sample

Between July 2017 and January 2019, 337 cancer patients received MAID in Alberta. Of these patients, 193 (57.3%) completed at least one PROs questionnaire in the year prior to their death, while 144 (42.7%) had no questionnaires. Table 1 presents the demographic and clinical factors for the full cohort and the subgroups with and without PROs data. The PROs group was significantly younger at the time of MAID provision than the group without PROs. The most common tumour group in both subgroups was gastrointestinal, followed by intrathoracic for the PROs group and “other” for the group without PROs. The group without PROs was significantly more likely to have a CCI of 1 or higher, indicating a greater prevalence of comorbidities. The median number of completed questionnaires was 4 (IQR = 1–7; rounded to the nearest whole numbers, as only complete questionnaires were included).

### 3.2. Time between MAID Request and Provision Dates

The vast majority of the total cohort (*n* = 294, 87.6%) made the request for MAID in the three months prior to their provision date. Of these, 224 requests (76.2%) were made within the same month that MAID was provided. The median time (in months) from MAID request dates to actual provision dates, calculated for the full cohort, was 1 month (IQR = 1–2). Table 2 presents the detailed distribution.

### 3.3. Symptom Trajectories

#### 3.3.1. ESAS-r Trajectories

**Figure 2:**
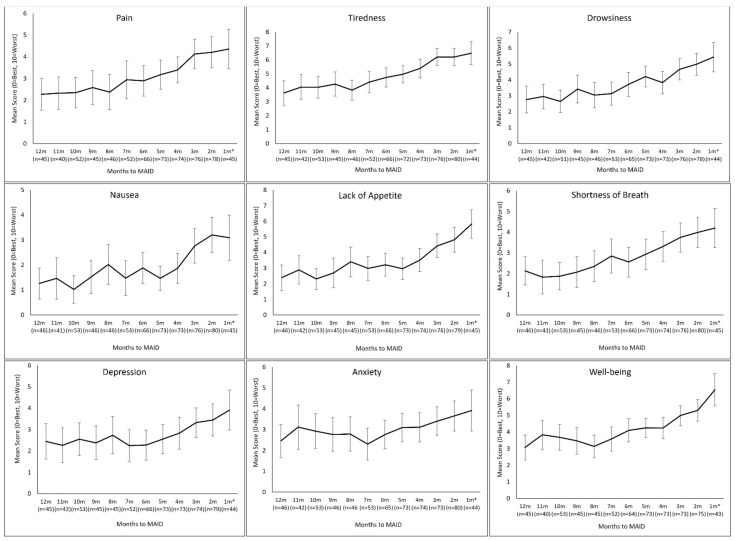
Trajectory of mean symptom scores in the 12 months prior to MAID, for each of the nine ESAS-r symptoms, as calculated using the mixed effects model. *n* = number of PROs questionnaires with a score (from 0–10) for each symptom indicated each month.

For the model selection, we examined the model fit criteria (AIC) of some common covariance structures (including compound symmetry, AR(1), scaled identity, unstructured and Toeplitz), as well as the mixed effect model with random effects. We selected the model with random intercept and slope as it had the smallest AIC. Nine MEMs were run, each with a single ESAS-r symptom as the dependent variable. Independent variables in each model included month before MAID (ranging from 12 to 1), age at MAID provision, sex, tumour group, CCI and neighborhood-level income (Figure 2). The first MEM used pain scores as the dependent variable. A significant main effect of time was noted (F = 49.9, *p* < 0.01, β = 0.222, 95% CI: 0.160–0.284), indicating an accelerated increase in pain scores closer to patients’ MAID provision dates, after accounting for the confounding effect of the covariates. Mean pain scores rose from just under 2.5 (out of a possible maximum severity of 10) at 12 months before MAID to just under 4.5 within the month MAID was provided. The same increasing trend, with a significant main effect of time, was observed with the other eight symptoms on the ESAS-r.

The largest increases in severity, from 12 months to 1 month, occurred in tiredness, lack of appetite, and well-being (higher scores indicate a worse state of well-being), with increases of around 3. The smallest increases occurred in depression and anxiety, with increases of just over 1. The number of questionnaires included at each time point is indicated on the figure. Table 3 lists detailed statistics from the MEM for each symptom.

#### 3.3.2. Symptom Complexity Level Trajectory

**Figure 3:**
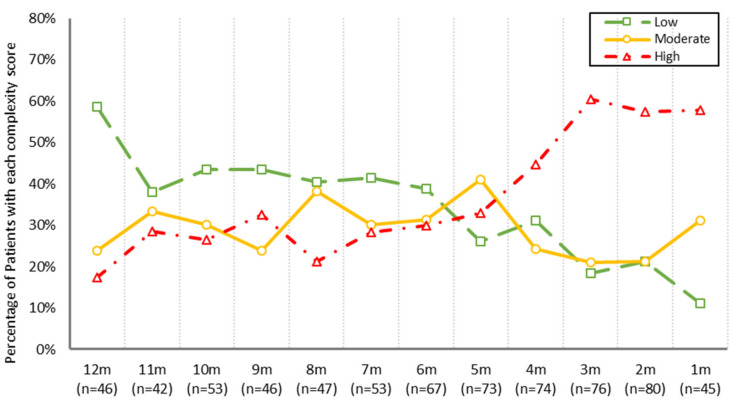
Distribution of symptom complexity in the cohort with PROs information in the 12 months prior to MAID provision. *n* = number of PROs questionnaires completed each month. The percentage of patients each month with high symptom complexity is shown in red (triangles), moderate symptom complexity in yellow (circles), and low symptom complexity in green (squares).

The 193 patients with PROs data completed 922 questionnaires in total. Of these, 336 questionnaires (36.4%) were classified as low complexity, 262 (28.4%) as moderate complexity, and 324 (35.1%) as high complexity. Results of the GEE showed that symptom complexity levels increased significantly over time (OR = 1.20 (95% CI: 1.14–1.23), SE = 0.028, Wald χ^2^ = 42.0, *p* < 0.01), as patients approached their MAID provision dates. Figure 3 depicts the distribution of symptom complexity levels by month prior to MAID. Less than 20% of patients had high complexity at 12 months prior to MAID and about 60% of patients had low complexity. There is a noticeable change just one month later, at month 11, as the share of low complexity patients drops below 40%, while moderate and high complexity increase. Another noticeable change takes place after month 5, with the share of moderate and low complexity patients decreasing and high complexity patients sharply increasing. During the last month of life, nearly 60% of patients experienced high symptom complexity, while only 10% experienced low complexity.

## 4. Discussion

This descriptive study utilized retrospective, routinely collected administrative and PROs data to examine how symptoms and symptom complexity changed over the last year of life in a cohort of cancer patients who received MAID. The findings provide clinically relevant information about this cohort of advanced cancer patients. To our knowledge, this is the first study to evaluate longitudinal changes in self-reported symptoms in a cohort of cancer patients who received MAID.

### 4.1. Increased Symptom Burden and Complexity Prior to MAID Provision

Our findings are generally consistent with available evidence suggesting that symptoms change at different rates and at different points in time among cancer patients, and the escalation of symptoms often accelerates closer to the end-of-life [30,31]. Specifically, our findings and the literature suggest that symptom severity increases most sharply around four months prior to death [30,32]. Patients in this study who completed CCA’s routine PROs questionnaire experienced progressive increases in all nine symptoms reported on the ESAS-r over the 12 months prior to MAID. In addition to specific symptoms increasing, the patient cohort also experienced considerable changes in symptom complexity levels. The observed symptom complexity of the cohort, presented in Figure 3, can be viewed as three distinct phases. First, month 12 can be thought of as the baseline, or the typical distribution of symptom complexity in CCA. At any given time in CCA, looking at the total patient population, about 15% of patients have high complexity, 25% have moderate complexity, and 60% have low complexity [33]; we see this distribution at month 12. Months 11 through 5 can be thought of as the second phase, where low complexity remains near or below 40%, and moderate and high complexity alternately increase and decrease. After month 5, the third phase occurs, where moderate and low complexity decrease (with a steep increase in moderate complexity occurring in the month of MAID provision) and high complexity sharply increases.

Although in the clinical setting symptom management and supportive care decisions must be informed by each patient’s unique set of symptoms at a given moment, understanding that there are distinct symptom complexity phases in the last 12 months of life could benefit clinical teams. Identifying these phases provides insight into when end-of-life conversations, including goals of care, advanced care planning, and the possibility of discontinuation of disease-directed treatments, should be incorporated into clinical conversations. Additionally, these time points could serve as flags for clinicians to initiate additional conversations with patients about symptom management and associated resources that may be available.

While the escalating symptom complexity scenario observed in our data is not surprising for patients with advanced cancer [30], the complexity in the last four months contextualizes the worsening health status of these patients and may help explain why they received MAID so soon after making their written request. The majority (87.6%) received MAID within three months following their request, the same time period during which symptoms and complexity were highest. Further, 66.7% of MAID procedures occurred within the same month as the request, possibly reflecting the magnitude of personal suffering related to elevated symptoms and complexity. Although this study did not set out to understand how patients’ ambulatory cancer care teams addressed the escalating symptoms reported by the patient on their PROs questionnaire, further research into the corresponding interventions, referrals and end-of-life conversations that occurred during this period should be considered in the future.

### 4.2. Implications for Practice

The findings point to the importance of routinely utilizing and collecting PROs with all cancer patients, and perhaps especially with patients who are living with advanced cancer or patients in palliative care. This study was possible because many patients in the MAID cohort completed CCA’s routine PROs questionnaire at least once in the last year of life, enabling us to track their symptom trajectories over time. No such data are available for patients who did not participate in this structured approach to self-reporting their symptoms and concerns. The subgroup of 144 patients that did not complete PROs questionnaires was older and had significantly more comorbidities than the PROs cohort. Studies have shown that patients report more symptoms on screening tools than they verbally report to their care team [34]; with this in mind, patients who do not complete a screening tool are at risk of underreporting their symptoms, and in turn having their concerns unaddressed. The care teams of these patients may have had limited visibility of the symptoms and associated symptom complexity they were experiencing. Without PROs information, it is difficult, if not impossible, to know the full extent of suffering that patients are experiencing.

Utilizing a symptom complexity level alongside detailed symptom scores may provide the clinical team a useful flag in the advanced cancer population. The literature suggests that palliative care is often initiated late for patients receiving treatment for advanced cancer [35]. A recent American study that aimed to explore the actual and missed opportunities for end-of-life care discussions with advanced cancer patients in ambulatory care found that only 5% of encounters included documented end-of-life discussions, while 38% of encounters revealed missed opportunities for these discussions [36]. As a result of such evidence, organizations such as the National Comprehensive Cancer Network [37] and the Canadian Hospice Palliative Care Association [38] recommend that cancer providers initiate early discussions about goals of care with cancer patients who have a life expectancy of less than one year. However, with the introduction of new therapeutic agents to control the progression of advanced cancer [39], understanding when a patient has entered the last year of life has become more challenging [40]. As a result of the findings of the current study, further research should be done to identify if the escalation of symptom complexity (both moderate and high) occurs within a similar timeframe in the broader advanced cancer population. If this was so, changes in symptom complexity may provide a valuable tool to identify when patients are entering the last year of life and, therefore, when end-of-life conversations should be consistently integrated into clinical encounters within ambulatory oncology. Clinicians and care teams could also draw on these findings to support the closer monitoring of PROs information once a patient’s symptom complexity level is classified as high. When patients are flagged as having high symptom complexity, careful attention should be paid to key symptoms such as tiredness and lack of appetite, which showed the largest changes in mean scores in the last year of life.

Finally, when trying to ascertain why individuals may choose to receive MAID, physical symptom burden and a loss of meaning, autonomy and identity [7] have been identified as predictors. A widely accepted approach to minimizing these types of distress includes the early integration of a palliative approach to care [38]. Although we did not examine our cohort’s use of palliative care services in this study, as these clinical data were largely unavailable, the 2019 annual report on MAID in Canada suggests that 82% of all MAID patients in Canada accessed palliative services, and most others were offered this service and declined it [10]. Within the ambulatory setting, there is wide recognition that the integration of palliative and supportive care services into routine cancer care is required to better meet the supportive care needs of this population [41,42], but data supporting this as routinely occurring in practice are scarce.

### 4.3. Study Limitations

This study provides new contributions to the Canadian literature on MAID; however, several limitations should be noted. First, the data used in this study were collected from routine clinical care encounters within CCA. The completion of the PROs questionnaire was a reflection of patient participation in routine care practices, resulting in 42.7% of the MAID cohort not having PROs data during the last year of their life. The mixed effects model was selected as the best method to manage the missing data, but has the potential for bias, if data are not missing at random as we assumed; this is always a risk in observational studies of this type [43]. Additionally, as the date of diagnosis was not included in the dataset, some patients may have received their initial diagnosis within, rather than prior to, the 12-month study period. We can only be sure that all patients had cancer at the time of completing each PROs questionnaire, and that all patients indicated cancer as their primary condition on their written MAID request. As more than 40% of the total MAID cohort was excluded from the symptom trajectory analysis due to missing PROs data, the estimations of symptom burden and complexity in the cohort may have been somewhat under or overestimated. Finally, the findings must be interpreted with caution, as firm conclusions cannot be drawn without including a cohort of patients with cancer who died naturally (without the use of MAID) for comparison.

### 4.4. Future Directions for Research

As mentioned, this is the first of several studies currently being conducted with this cohort of cancer patients who received MAID. This first study provides a descriptive look into the symptom burden and complexity trends over the last 12 months of life. Our next step will be to include a matched cohort of cancer patients who died in the same time period (without receiving MAID) and compare the two groups. Further research exploring clinical responses to symptom complexity is in the design phase. All studies will contribute to the Canadian literature on MAID and provide insight into advanced cancer patients’ experiences in the last 12 months of life. This information could be helpful to ambulatory cancer care teams, MAID practitioners and other health care providers, including general practitioners, palliative care specialists and psychosocial oncology providers.

## 5. Conclusions

This study helps characterize the symptom profile of cancer patients who are provided MAID in Alberta, and the exploration extends our understanding of the symptom burden experienced at the end-of-life. The individual symptoms and overall symptom complexity of the cohort increased over the 12-month period, with three distinct phases of symptom complexity, and the last four months of life appeared particularly challenging. These findings could serve to highlight patterns in key symptoms and symptom complexity, and the resulting opportunities to focus clinical care and discussions with patients, families or caregivers on their concerns and possible interventions, such as end-of-life care. It is important to continue exploring the characteristics and symptoms of patients who request and receive MAID, to gain additional understanding about this population and how patients could be better supported prior to their death.

## Figures and Tables

**Table 1:** Patient characteristics for full cohort and subgroups with and without PROs information.

Characteristics	Full Cohort (*n* = 337)	Subgroup with PROs (*n* = 193)	Subgroup without PROs (*n* = 144)	*p* (PROs vs. No PROs)
Age at MAID provision (in years)				0.000
Mean (Min, Max, SD)	72.6 (26, 98, 12.0)	68.6 (26, 95, 11.5)	78.0 (50, 98, 10.6)	
Sex				0.788
Female	162 (48.1%)	94 (48.7%)	68 (47.2%)	
Male	175 (51.9%)	99 (51.3%)	76 (52.8%)	
Tumour groups				0.168
Breast	34 (10.1%)	17 (8.8%)	17 (11.8%)	
Gastrointestinal	86 (25.5%)	52 (26.9%)	34 (23.6%)	
Genitourinary	42 (12.5%)	21 (10.9%)	21 (14.6%)	
Gynecology	32 (9.5%)	22 (11.4%)	10 (6.9%)	
Hematology	29 (8.6%)	20 (10.4%)	9 (6.3%)	
Intrathoracic	53 (15.7%)	33 (17.1%)	20 (13.9%)	
Other ^a^	61 (18.1%)	28 (14.5%)	33 (22.9%)	
CCI				0.001
0	200 (59.3%)	129 (66.8%)	71 (49.3%)	
≥1	137 (40.7%)	64 (33.2%)	73 (50.7%)	
Neighborhood income (CAD$)				0.001
Median	97,230	103,179	88,517	

^a^: “Other” includes Central Nervous System, head and neck, melanoma, non-melanoma skin, sarcoma, and “other primary” cancers.

**Table 2:** Time from MAID request dates to provision dates for full cohort (*N* = 337).

Number of Months	*n*	%
≥10 month	4	1.2
7–9 months	6	1.8
4–6 months	32	9.5
3 months	15	4.5
2 months	55	16.4
1 month ^a^	224	66.7

^a^: “1” indicates that the request occurred within the same month as MAID provision.

**Table 3:** Results from nine mixed effects models, presenting the main effect of time on each individual ESAS-r symptom, controlling for confounding factors (age at MAID provision, sex, tumour group, CCI and neighborhood-level income).

Symptoms	Β ^a^ (95% CI)	SE ^b^	*F*	*p*
Pain	0.222 (0.160–0.284)	0.031	49.9	0.000
Tiredness	0.250 (0.194–0.306)	0.029	77.0	0.000
Drowsiness	0.217 (0.157–0.277)	0.031	50.6	0.000
Nausea	0.170 (0.114–0.226)	0.028	35.6	0.000
Lack of appetite	0.250 (0.182–0.319)	0.035	52.0	0.000
Shortness of breath	0.168 (0.112–0.224)	0.029	34.8	0.000
Depression	0.137 (0.082–0.193)	0.028	24.0	0.000
Anxiety	0.060 (0.004–0.116)	0.028	4.47	0.035
Well-being	0.223 (0.165–0.280)	0.030	58.0	0.000

^a^: B = beta (estimate); a positive beta indicates that as the value of the independent variable increases, the mean of the dependent variable also tends to increase. ^b^: SE = Standard Error.

## Data Availability

The data used in this study are not publicly available; however, they may be available upon request. Please contact the corresponding author.
